# Quality of patient-reported outcome measures for primary dysmenorrhea: a systematic review

**DOI:** 10.1007/s11136-023-03517-8

**Published:** 2023-10-30

**Authors:** Katharina Piontek, Michaela Gabes, Gesina Kann, Marie Fechtner, Christian Apfelbacher

**Affiliations:** Institute of Social Medicine and Health Systems Research, Medical Faculty, Otto-von-Guericke University Magdeburg, Leipziger Str. 44, 39120, Magdeburg, Germany

**Keywords:** Primary dysmenorrhea, Patient-reported outcome measures, Questionnaire, Psychometric properties

## Abstract

**Purpose:**

To conduct a systematic review of the quality of patient-reported outcome measures (PROMs) for primary dysmenorrhea (PDys) using the COnsensus-based Standards for the selection of health Measurement INstruments (COSMIN) methodology, and to derive recommendations for use of the PROMs.

**Methods:**

We searched PubMed and Web of Science for studies reporting on the development and/or validation of any PROMs for women with PDys. Applying the COSMIN Risk of Bias Checklist, we assessed the methodological quality of each included study. We further evaluated the quality of measurement properties per PROM and study according to the criteria for good measurement properties, and graded the evidence. Based on the overall evidence, we derived recommendations for the use of the included PROMs.

**Results:**

Data from seven studies reporting on four PROMs addressing different outcomes were included. Among those, the Adolescent Dysmenorrhic Self-Care Scale (ADSCS) and the on-menses version of the Dysmenorrhea Symptom Interference Scale (DSI) can be recommended for use. The Exercise of Self-Care Agency Scale (ESCAS) and the Dysmenorrhea Daily Diary (DysDD) have the potential to be recommended for use, but require further validation. The off-menses version of the DSI cannot be recommended for use.

**Conclusions:**

The ADSCS can be recommended for the assessment of self-care behavior in PDys. Regarding measures of impact, the on-menses version of the DSI is a suitable tool. Covering the broadest spectrum of outcomes, the DysDD is promising for use in medical care and research, encouraging further investigations. Further validation studies are indicated for all included PROMs.

**Supplementary Information:**

The online version contains supplementary material available at 10.1007/s11136-023-03517-8.

## Plain English summary

Primary dysmenorrhea (PDys), defined as menstrual pain in the absence of pelvic pathology, is among the most common gynecological conditions among women of reproductive age. To assess patient-reported outcomes (PROs) related to PDys, several disease-specific patient-reported outcome measures (PROMs) are applied. An evaluation of the quality of PROMs for PDys using a standardized methodology is currently not available, but would help researchers and clinicians to select the most suitable instrument. We aimed (a) to conduct a systematic review of the quality of PROMs for PDys using the COnsensus-based Standards for the selection of health Measurement INstruments (COSMIN) methodology, and (b) to derive recommendations for their use in research and patient care. Data from seven studies reporting on four PROMs focusing on various outcomes were included. Among the identified instruments, the Adolescent Dysmenorrhic Self-Care Scale (ADSCS) measuring self-care behavior, and the on-menses version of the Dysmenorrhea Symptom Interference Scale (DSI) assessing the impact of PDys on physical activities, sleep, daily activities, work, leisure and social activities, and mood can be recommended for use. The Dysmenorrhea Daily Diary (DysDD) assessing menstrual bleeding, pelvic pain, use of rescue medication, and impact of pelvic pain/cramps on daily life does currently not fulfill the COSMIN criteria for a recommendation. However, as the tool is capturing the broadest spectrum of outcomes, it appears promising for use in research and patient care, and further investigations are encouraged. The off-menses version of the DSI cannot be recommended for use.

## Background

Primary dysmenorrhea (PDys), defined as menstrual pain in the absence of any organic cause [1], is among the most common gynecological conditions among women of reproductive age [2]. The prevalence of PDys ranges from 45 to 95% among menstruating women, whereby up to 29% experience severe pain [3]. The burden of PDys is substantial with negative impact on physical and mental health, physical activity, school and work productivity, sleep, and health-related quality of life [4]. Treatment commonly involves drugs, medicinal plants, and acupressure [5]. Evaluating the efficacy of these interventions from the patients’ perspective is critical, and patient-reported outcome measures (PROMs) are suitable tools for this purpose [6]. When selecting an instrument, the construct of interest and the quality of measurement properties of available tools should be taken into account. The COnsensus-based Standards for the selection of health Measurement INstruments (COSMIN) methodology [7] provides a profound framework for the assessment of the methodological quality of single studies on measurement properties of PROMs, and for the evaluation of the quality of measurement properties of PROMs. The COSMIN methodology has been specifically developed to guide the selection of PROMs in research and clinical practice in an international Delphi study involving experts with backgrounds in epidemiology, statistics, psychology, and clinical medicine [8]. COSMIN provides a methodological approach including detailed, standardized, and transparent criteria, and practical tools for selecting the most appropriate instrument [9].

A systematic review of disease-specific PROMs for PDys and an assessment of the quality of their psychometric properties is currently not available, but would facilitate the selection of the most appropriate instrument for researchers and clinicians. Using the COSMIN methodology, we pursued the following aims:

1. To conduct a systematic review of the quality of existing disease-specific PROMs for PDys, i.e.,
  i. to evaluate the quality of development and/or validation studies
  ii. to evaluate the psychometric properties of the identified PROMs including aspects of interpretability and feasibility
  iii. to grade the evidence
2. To derive recommendations for use of the identified PROMs in research and patient care.

## Methods

### Protocol and registration

The present systematic review was conducted following the recommendations of the Preferred Reporting Items for Systematic Reviews and Meta-Analyses Protocols (PRISMA-P) statement [10] and the COSMIN guideline and manual for systematic reviews of PROMs [7, 11]. The protocol has been registered in the International Prospective Register of Systematic Reviews (PROSPERO) (CRD42022358458).

### Literature search

Using the databases PubMed and Web of Science, a systematic search of the literature for studies on the development and/or validation of any PROMs for PDys was performed on 12 September 2022. Details on the search strategy including search elements and syntax for search in PubMed are displayed in Appendix 1. An update of our literature search was conducted on 28 June 2023.

### Eligible studies

Inclusion and exclusion criteria are displayed in Table 1.

**Table 1:** Inclusion and exclusion criteria

	Inclusion criteria	Exclusion criteria
Population	Women with primary dysmenorrhea	Women with other urological and/or gynecological diseases of the lower abdomen
Study design	PROM development and/or validation study	All other study designs
Outcome	All patient-reported outcomes	Non patient-reported outcomes, e.g., biomarkers, laboratory data
Type of measurement instrument	PROM	All others
Publication type	Articles with available full text	Abstracts

*PROM* patient-reported outcome measure

### Study selection

Following deduplication of the records in Citavi 6, we performed the screening of titles and abstracts using Rayyan [12]. To assess initial eligibility, titles and abstracts were evaluated according to the inclusion and exclusion criteria independently by two reviewers. For articles considered eligible at this stage, the full texts were searched and also evaluated independently by two reviewers according to the predefined criteria. In case of any disagreement, consensus was reached within the research team.

### Evaluation of measurement properties

All measurement properties were evaluated according to the COSMIN manual (based on [7, 11, 13]) following three sub steps as outlined below. Data collection forms and details from data extraction are available from the corresponding author upon reasonable request.

The following measurement properties were assessed:

- Content validity.
- Internal structure including structural validity, internal consistency, and cross-cultural validity/measurement invariance.
- Remaining measurement properties including reliability, measurement error, criterion validity, hypotheses testing for construct validity, and responsiveness.

#### Assessment of the methodological quality of the included studies

The methodological quality of each single study on a measurement property was evaluated independently by two reviewers with psychological background and experience in the application of the COSMIN methodology using the COSMIN Risk of Bias checklist [11]. The COSMIN Risk of Bias checklist consists of 10 boxes encompassing all standards needed to assess the quality of a study on that specific measurement property (Appendix 2). Content validity is considered the most important measurement property, and the available evidence from content validity studies and the PROM development study was considered for the evaluation of content validity. The assessment is based on five items on relevance, one item on comprehensiveness and four items on comprehensibility. The content validity is also rated by the reviewers themselves, and their ratings are considered as additional to the evidence from the literature. However, if no content validity studies are available, or only content validity studies of inadequate quality, and the PROM development is of inadequate quality, the rating of the reviewers determines the overall ratings [13]. The methodological quality of the studies was rated on a four-point rating scale as either very good, adequate, doubtful, or inadequate. The overall quality of a study was determined by the lowest rating of any standard in the box (“worst score counts”) [11].

#### Assessment of the quality of measurement properties

The quality of measurement properties was assessed by one reviewer, and a second reviewer evaluated 20% of the included data for quality assurance purposes. The result of each single study on a measurement property was evaluated against the criteria for good measurement properties, and rated as either sufficient ( +), insufficient ( −), or indeterminate (?) (Appendix 3). We further summarized the quality of the evidence per measurement property per PROM, and the summarized results were also rated against the criteria for good measurement properties. Additionally, we extracted data on interpretability and feasibility of the PROMs. These aspects are not formally evaluated by the COSMIN tools, but are viewed as important considerations for the practical use of a measurement instrument (see [14] for details).

#### Grading the evidence

The quality of evidence of the summarized results was graded using the Grading of Recommendations Assessment, Development and Evaluation (GRADE) approach [14]. In case of concerns regarding the trustworthiness of a result, the quality of evidence is downgraded per measurement property per PROM. Downgrading was possible due to risk of bias (methodological quality of studies assessed by the RoB checklist), inconsistency (unexplained inconsistency of results across studies), imprecision (total sample size of available studies), and/or indirectness (evidence from different populations than the population of interest). The quality of evidence was rated as either high, moderate, low, or very low. We did not grade the quality of evidence if an overall rating was indeterminate or inconsistent. To generate recommendations for use of the identified PROMs, we categorized each instrument as follows [7]:

A. PROMs with evidence for sufficient content validity (any level) and at least low-quality evidence for sufficient internal consistency.
B. PROMs categorized not in A or C.
C. PROMs with high-quality evidence for an insufficient measurement property.

PROMs of category A can be recommended for use, while PROMs of category B have the potential to be recommended for use, but require further validation. PROMs of category C should not be recommended for use.

## Results

### Literature search

**Fig. 1:**
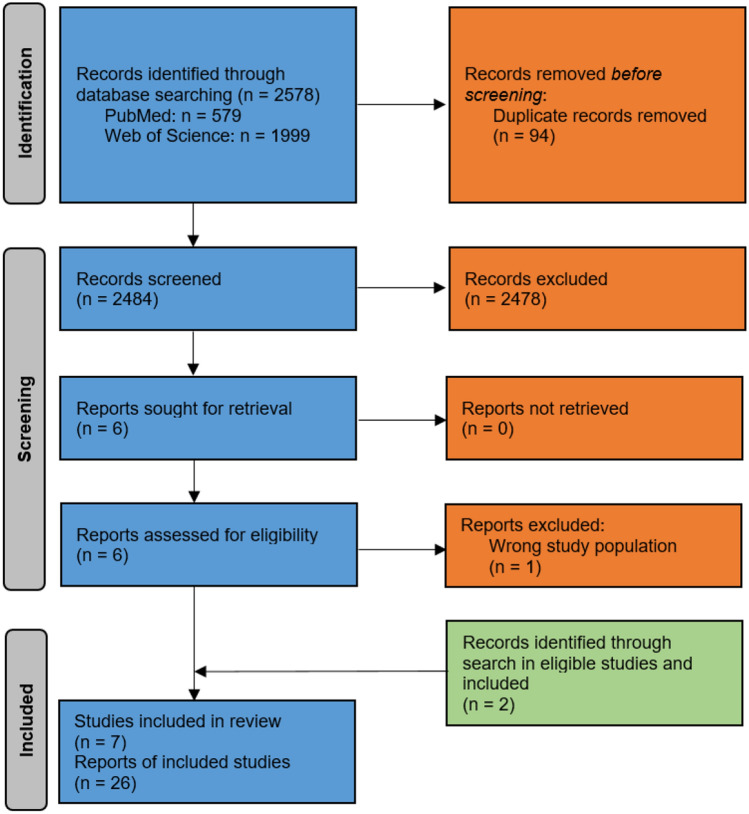
Adapted preferred reporting items for systematic reviews and meta-analyses (PRISMA) 2020 flow diagram

The results of our literature search are displayed in Fig. 1. For data extraction, we included seven studies reporting on four different PROMs. Two studies reported on the Dysmenorrhea Daily Diary (DysDD) [15, 16], and one study, respectively, reported on the Exercise of Self-Care Agency Scale (ESCAS) [17], the Adolescent Dysmenorrhic Self-Care Scale (ADSCS) [18], and on the Dysmenorrhea Symptom Interference Scale (DSI) [19]. The studies on the ESCAS and the ADSCS referred to the respective development study [20, 21], which we searched and considered for evaluation of the content validity of these instruments.

Additionally, we considered a review of self-reported pain and symptom measures for PDys [22], and evaluated the included tools regarding eligibility. The identified instruments did not meet our predefined criteria and were excluded (Appendix 4). The update of our literature search did not yield new eligible studies.

### Characteristics of the included PROMs and study populations

Details of the included PROMs and study populations are presented in Tables 2 and 3. The purpose of the ESCAS and ADSCS is to assess self-care behavior using 43 and 35 items, respectively, which are rated on a 5-point (ESCAS) and 6-point (ADSCS) Likert scale. The DSI is measuring the impact of PDys on physical activities, sleep, daily activities, work, leisure and social activities, and mood. The instrument comprises nine items, which are rated on a five-point Likert scale with one version each for on-menses and off-menses using different recall periods (last 24 h vs. last menstrual period). The DysDD is conceptualized as daily diary aiming to assess menstrual bleeding, pelvic pain, use of rescue medication, and impact of pelvic pain or cramps on daily life using 10 items, which are scored independently on different scale formats.

**Table 2:** Characteristics of the included instruments

	ESCAS	ADSCS	DSI	DysDD
Construct	Self-care behavior	Self-care behavior	Impact of dysmenorrhea on physical activities, sleep, daily activities, work, leisure and social activities, mood	Menstrual bleeding, pelvic pain, use of rescue medication, impact of pelvic pain/cramps on daily life
Target population	Adolescent girls with PDys	Adolescent girls with PDys	Adolescent girls and women with PDys	Women with PDys
Mode of administration	Self-administered	Self-administered	Self-administered	Self-administered
Recall period	4 weeks	4 weeks	on-menses version: 24 h; off-menses version: last menstrual period	24 h
(Sub)scales (number of items)	43 items; four dimensions: Active vs. passive response to situations: 12 items; Motivation: 9 items; Knowledge base: 9 items; Sense of self-worth: 13 items	35 items; two dimensions: *Externally oriented behaviors:* Searching for knowledge (4 items), expression of emotions (6 items), seeking assistance (3 items), control over external factors (7 items) *Internally oriented behaviors:* Resource utilization (10 items), self-control being (5 items)	0 subscales (9 items)	0 subscales (10 items)
Response options	5-point Likert scale: 0 (very uncharacteristic of me) to 4 (very characteristic of me)	6-point Likert scale: 1 (totally disagree) to 6 (totally agree)	5-point Likert scale: 1 (not at all) to 5 (very much)	*Item 1 (menstrual bleeding):* 5-point Likert scale: 0 (no bleeding) to 5 (heavy bleeding) *Item 2 (use of sanitary protection):* 4-point Likert scale: 0 (no pieces) to 3 (3 or more pieces) *Item 3 (pelvic pain or cramps):* Numeric rating scale: 0 (no pain or cramps) to 10 (extreme pain or cramps) *Item 4 (use of rescue medication):* dichotomous scale (yes/no) *Item 5 (amount of rescue medication):* continuous score (#pills) *Item 6 (impact on work/school):* 5-point Likert scale: 0 (not at all) to 5 (extremely) *Item 7 (hours of missed work/school:* continuous score (hours and minutes) *Items 8–10 (impact on physical activities, impact on social/leisure activities, impact on sleep):* 5-point Likert scale: 0 (not at all) to 5 (extremely)
Range of scores/scoring	0–172	40–240	9–45	All items are scored independently
Original language	English	English	English	English
Available translations	Chinese–Cantonese	Chinese–Cantonese	Turkish	n/a

*ADSCS* adolescent dysmenorrhic self-care scale, *ESCAS* exercise of self-care agency scale, *DSI* dysmenorrhea symptom interference scale, *DysDD* dysmenorrhea daily diary, *PDys* primary dysmenorrhea

**Table 3:** Characteristics of the included study populations

Instrument	Reference	Sample size	Age in years; mean (*SD*) and/or median	Setting	Country (Language)	Measurement properties
ESCAS	Kearney and Fleischer 1979 [20]	*N* = 153	Not reported	Nursing students	USA (American English)	PROM development, content validity, reliability, hypotheses testing
	Wong et al. 2012a [17]	*N* = 477	M = 16.03 (SD = 1.57), range: 13–19	Secondary schools	Hong Kong (Chinese Cantonese)	Content validity, structural validity, internal consistency, reliability
ADSCS	Hsieh et al. 2004 [21]	*N* = 361	M = 15.5 (SD = 1.3), range: 13–18	High and senior high schools	Taipei Country (Taiwanese, Mandarin)	PROM development, structural validity, internal consistency, hypotheses testing
	Wong et al. 2012b [18]	*N* = 396	M = 15.8 (SD = 1.55)	Secondary schools	Hong Kong (Chinese Cantonese)	Content validity, structural validity, internal consistency, reliability, hypotheses testing
DSI	Chen et al. 2021 [19]	*Development study:* *N* = 30	*Development study:* M = 24.0 (SD = 6.3), range: 14–42	Survey panel registrants	USA (American English)	PROM development, content validity, structural validity, internal consistency, reliability, hypotheses testing, responsiveness
		*Validation study:* *N* = 686	*Validation study:* On-menses: M = 28.6 (SD = 6.9) Off-menses: M = 27.6 (SD = 8.1)			
DysDD	Nguyen et al. 2015 [15]	*For item generation:* *N* = 52 (including a subset of *n* = 12 women with comorbid pelvic pain condition (PPC))	*For item generation (n = 52):* 24 adolescents: M = 15.9 (SD = 1.2), range 14–17; 28 adults: M = 35.5 (SD = 9.2), range 18–49	Clinical setting	USA (American English)	PROM development
		*Pilot test:* *N* = 24	*Pilot test (n = 24):* 12 adolescents: M = 15.7 (SD = 1.1), range 14–17); 12 adults: M = 29.3 (SD = 9.7), range 18–44			
	Nguyen et al. 2017 [16]	*N* = 355	M = 29.0 (SD = 8.0), range 18–49	Clinical trial	Different countries, presumably English language, but not detailed: European Union, Australia, New Zealand, South America, Mexico, South Africa	Reliability, hypotheses testing, responsiveness

*M* mean, *SD* standard deviation, *ADSCS* adolescent dysmenorrhic self-care scale, *ESCAS* exercise of self-care agency scale, *DSI* dysmenorrhea symptom interference scale, *DysDD* dysmenorrhea daily diary

The sample sizes of the included studies ranged from 24 to 686 patients, and the overall age range was 13–49 years.

### Information on interpretability and feasibility

No data regarding interpretability and feasibility were reported for the ESCAS. For the on-menses and off-menses versions of the DSI, distribution-based minimal important difference (MID) estimates ranging from 0.27 to 0.36 were reported. Further, the anchor-based estimate was 0.28 for minimally important improvement and 0.18 for minimally important worsening. For the DysDD, data on the distribution of scores in the study population, missing data, and data on MID were provided. Within the framework of the development study [15], preliminary quantitative analyses were conducted showing a good distribution of responses with no major ceiling or floor effects and all response options utilized. Subsequent validation analyses [16] revealed that the items showed a good distribution of responses across response options at baseline. Furthermore, the majority of responses on day − 1 (the day before the initiation of menstrual bleeding) and on day 3 were concentrated at the lower end of the scale, whereas the responses on days 1 and 2 were grouped toward the higher end of the scale. At treatment cycle 2, the response distributions were comparable with baseline scores, with a general trend to show slightly lower scores, which was accompanied by lower mean scores for rescue medication items. All items of the DysDD showed floor effects at day  − 1, and the majority of items (items 3, 6, 8, and 9) then showed ceiling effects over days 1–2. The item assessing impact on sleep (item 10) did not show any ceiling effects, but floor effects on days − 1, 1, and 3. Analyses on missing data revealed that four participants (17%) missed one or more days of completing the DysDD during the pilot test. In the validation study, only women with complete data were included, and missing data were not imputed or carried forward for validation analyses. With respect to MID, analyses indicate that changes on the pelvic pain score (score range 0–10) of three points can be considered clinically meaningful. For all included PROMs, no data were available regarding scores and change scores for relevant subgroups and response shift.

Concerning feasibility, no study reported difficulties regarding the patient’s comprehensibility and administration of the PROM. Pretesting the ADSCS showed that it took 5–10 min to complete the questionnaire. The DysDD was administered as eDiary using a hand-held, electronic, touch-screen device. In the pilot test, participants found the format and functionality of the eDiary device easy to use and to incorporate into their daily lives [15]. Information on access to all identified PROMs is given in Appendix 5.

### Measurement properties of instruments

When evaluating the quality of the included studies using the COSMIN Risk of Bias checklist, the reviewers had a mean agreement of 81.4% across all studies. Major disagreements were resolved by discussion with a third reviewer having expertise with the COSMIN methodology.

#### Evaluation of content validity

The overall ratings of the PROM development and content validity studies are displayed in Appendix 6. The development study of the ESCAS [20] was rated ‘inadequate’ since the instrument was not developed for the target population. The content validity study [17] received a ‘doubtful’ rating because detailed information about different aspects of the procedure were not provided. The development study of the ADSCS [21] was rated ‘doubtful’ due to methodological weaknesses regarding the collection and analysis of qualitative data for PROM design, and due to methodological weaknesses of the pilot test. Likewise, the content validity study of the ADSCS [18] was rated ‘doubtful’ because details of the methodological approach were not described. The development study of the DSI [19] received an ‘inadequate’ rating because the scale was developed based on research literature, and a sample representing the target population was not involved in the design of the instrument. Due to methodological shortcomings when asking patients about relevance, the content validity study of the DSI [19] was rated ‘doubtful.’ The development study of the DysDD [15] received a ‘doubtful’ rating since the qualification of the interviewers was not described. For the DysDD, a content validity study was not performed.

The overall content validity rating per PROM and the evaluation of the quality of evidence is displayed in Appendix 7. The content validity of the ESCAS was rated ‘indeterminate,’ and we therefore did not assess the quality of evidence. The ADSCS and the DSI showed sufficient content validity, and the quality of evidence was rated ‘moderate’ since at least one content validity study of doubtful quality was available, respectively. Also the DysDD showed sufficient content validity, but the quality of evidence was rated ‘low’ because only a PROM development study of ‘doubtful’ quality was available, and a content validity study was not performed.

As we found no high-quality evidence for insufficient content validity of any PROM, we subsequently assessed the remaining measurement properties of each PROM.

#### Evaluation of the remaining measurement properties

The results of the evaluation of the quality of studies on measurement properties and the rating of the methodological quality of the instruments are displayed in Table 4. Based on the five validation studies available in total, the methodological quality of 26 single studies on measurement properties was evaluated. No study analyzed cross-cultural validity/measurement invariance, measurement error, and criterion validity. Regarding the ADSCS, it is important to note that the development study resulted in a 40-item questionnaire, for which structural validity, internal consistency, and hypotheses testing were assessed [21]. Evaluating these measurement properties showed sufficient structural validity, but insufficient internal consistency, and sufficient construct validity (data not shown). In the validation study [18], the instrument was revised resulting in a 35-items version, for which we analyzed and report the psychometric properties. The summarized results per PROM and measurement property are depicted in Table 5.

**Table 4:** Quality of studies on measurement properties and methodological rating of the instruments

PROM	References	Methodological quality (rating^a,b^)				
		Structural validity	Internal consistency	Reliability	Hypotheses testing	Responsiveness
ESCAS	Wong et al. 2012a [17]	Very good (?)	Very good (?)	Adequate (+)		
ADSCS	Wong et al. 2012b [18]	Very good (+)	Very good (+)	Adequate (+)	Adequate (+)	
DSI	Chen et al. 2021 [19]					
On-menses		Very good (+)	Very good (+)	Doubtful (?)	Very good (+) Adequate (+)	Doubtful (±)
Off-menses		Very good (+)	Very good (+)		Very good (-) Adequate (-)	
DysDD	Nguyen et al. 2017 [16]			Adequate (-/-/+)	Adequate (+ /+) Doubtful (+)	Very good (+) Inadequate (+)

^a^No study has analyzed cross-cultural validity/measurement invariance, measurement error, and criterion validity

^b^Rating: ( +) sufficient, (−) insufficient rating, (?) indeterminate

*PROM* patient-reported outcome measure, *ADSCS* adolescent dysmenorrhic self-care scale, *ESCAS* exercise of self-care agency scale, *DSI* dysmenorrhea symptom interference scale, *DysDD* dysmenorrhea daily diary

**Table 5:** Overall rating of the quality of the measurement properties per instrument

PROM	Summary or pooled result	Overall rating	Quality of evidence
*ESCAS*			
Structural validity	Not all information for sufficient rating reported, sample size: 477	Indeterminate	–
Internal consistency	Alpha = 0.77–0.92, no evidence for sufficient structural validity, sample size: 477	Indeterminate	–
Reliability	ICC = 0.81, sample size: 477	Sufficient	Moderate (due to risk of bias)
*ADSCS*			
Structural validity	CFI = 0.96, sample size 396	Sufficient	High
Internal consistency	Alpha = 0.71–0.94, sample size 396	Sufficient	High
Reliability	ICC = 0.93, sample size: 53	Sufficient	Moderate (due to risk of bias)
Hypotheses testing	1 out of 1 hypothesis confirmed, sample size: 396	Sufficient	9a. Moderate (due to risk of bias)
*DSI*			
Structural validity	On-menses: CFI = 0.95, sample size: 260	Sufficient	High
	Off-menses: CFI = 0.96, sample size: 426	Sufficient	High
Internal consistency	On-menses: Alpha = 0.93 (Time 1) and 0.95 (Time 2), sample size: 260	Sufficient	High
	Off-menses: Alpha = 0.91, sample size: 426	Sufficient	High
Reliability	ICC or weighted Kappa not reported, sample size: 32 (on-menses)	Indeterminate	–
Hypotheses testing	On-menses: 6 out of 6 hypotheses confirmed, sample size: 260	Sufficient	9a. High
	Off-menses: 3 out of 5 hypotheses confirmed, sample size: 426	Insufficient	9a. High
Responsiveness	On-menses: 1 out of 1 hypothesis confirmed; 1 out of 2 hypotheses confirmed, sample size: 260	Inconsistent → Overall rating based on the two confirmed hypotheses (Sufficient)	10c. Moderate (due to inconsistency)
*DysDD*			
Reliability	Inner-cycle: Weighted Kappa = ≤ 0.2–0.5, sample size: 102	Insufficient	High
	Intra-cycle: Weighted Kappa = 0.7, sample size: 143	Sufficient	Moderate (due to risk of bias)
Hypotheses testing	76 out of 86 hypotheses confirmed, sample size: 335	Sufficient	9a. High 9b. Moderate (due to risk of bias)
Responsiveness	12 out of 12 hypotheses confirmed, sample size: 335	Sufficient	10a. High 10b. High

*CFI* comparative fit index, *ICC* intraclass correlation coefficient, *PROM* patient-reported outcome measure, *ADSCS* adolescent dysmenorrhic self-care scale, *ESCAS* exercise of self-care agency scale, *DSI* dysmenorrhea symptom interference scale, *DysDD* dysmenorrhea daily diary

#### Recommendation

The ADSCS and the on-menses version of the DSI were placed into category A (Table 6). The ESCAS and the DysDD were placed into category B, and the DSI off-menses version was placed into category C.

**Table 6:** Recommendations for use of the identified instruments

PROM	Category A		Category C	
	Sufficient content validity (any level)	At least low-quality evidence for sufficient internal consistency	High quality evidence for an insufficient measurement property	Recommendation according to COSMIN criteria
ESCAS	×	×	×	B
ADSCS	√	√	×	A
DSI				
On-menses	√	√	×	A
Off-menses	√	√	√	C
DysDD	√	×	×	B

Recommendation category A: Instrument can be used

Recommendation category B: Instrument has the potential to be used, but requires further validation

Recommendation category C: Instrument cannot be used

*PROM* patient-reported outcome measure, *COSMIN* COnsensus‐based standards for the selection of health measurement instruments, *ADSCS* adolescent dysmenorrhic self-care scale, *ESCAS* exercise of self-care agency Scale, *DSI* dysmenorrhea symptom interference scale, *DysDD* dysmenorrhea daily diary

## Discussion

This systematic review provides a synthesized evaluation of the quality of PROMs for PDys applying the COSMIN methodology. Among the four identified instruments, the ADSCS and the on-menses version of the DSI can be recommended for use in future research (COSMIN category A). We further found that the ESCAS and the DysDD have the potential to be recommended, but require further validation (COSMIN category B). The off-menses version of the DSI cannot be recommended for use (COSMIN category C). The identified PROMs address different outcomes, which is of importance for their application in research and clinical care.

The classification of a PROM into a recommendation category according to the COSMIN methodology is based on the evaluation of content validity and structural validity. Although the ADSCS and the on-menses version of the DSI meet the requirements for a recommendation according to these criteria, significant evidence gaps remain. All included PROMs show substantial conceptual and methodological flaws, which need to be discussed.

The ESCAS was developed to measure a person’s exercise of self-care agency based on Orem's self-care deficit nursing theory [23]. Most importantly, the ESCAS is a generic instrument for the assessment of self-care ability, and it was not designed for use in women with PDys. Due to methodological weaknesses of the validation study, which was performed in adolescent girls with PDys [17], we could not determine the content validity, and also structural validity and internal consistency could not be evaluated since the required data were not reported. Extending their work on the ESCAS, Wong and colleagues have translated and validated the ADSCS [18], which also aims to assess self-care behavior of adolescent girls with PDys. The development of the ADSCS involved a sample of the target population, and also a cognitive interview study was performed [21]. Data from the subsequent translation and validation study [18] showed sufficient content validity and sufficient internal consistency, indicating that the instrument can be recommended for use. However, since patients were not asked about comprehensiveness in the development phase, and relevance and comprehensiveness were not assessed from the patients’ perspective, further content validity assessments are indicated.

Applying the COSMIN criteria further suggests that the ESCAS has the potential to be recommended for use. Nevertheless, in view of its substantial methodological weaknesses and the availability of the ADSCS measuring the same construct with sufficient measurement properties, we oppose further validation of the ESCAS and consider the ADSCS as preferred measure of self-care behavior in PDys.

The DSI measuring the impact of PDys on various outcomes is available as version on-menses with a 24-h recall period, and as version off-menses referring to the last menstrual period [19]. We found sufficient content validity and sufficient internal consistency of both versions, indicating that the instrument can be potentially recommended for use. Concerning aspects of feasibility, data on MID are available, which is important for the application of the instrument by researchers and clinicians. Notably, the DSI was developed based solely on research literature, and a sample representing the target population was not involved in the design of the instrument. As patient participation is considered a major quality criterion for PROM development [24], the DSI is of insufficient quality in this regard. Moreover, construct validity is a concern of the off-menses version. Construct validity was determined by examining correlations of symptom interference with menstrual pain severity, perceived stress, and sleep disturbance referring to the last 24 h and to the last menstrual period for the on- and off-menses version, respectively. For the off-menses version, the observed correlations were not in accordance with the predefined hypotheses, which might be related to recall bias resulting from a potentially too long recall period. Consequently, the construct validity of the DSI off-menses version was rated ‘insufficient,’ and this version cannot be recommended for use.

Capturing the broadest spectrum of outcomes, the DysDD [16] was found to have sufficient content validity. Meeting the scientific and regulatory requirements for PROM development [25], the instrument was developed based on profound concept elicitation and comprehensive qualitative assessments in the target population, and also a cognitive interview study was performed. However, as data from content validity studies were are not available, the content validity of the DysDD was solely rated by the reviewers, resulting in low quality of evidence. These findings indicate that studies on the content validity of the DysDD are highly recommended. Another shortcoming of the DysDD concerns the lack of data regarding structural validity and internal consistency. Furthermore, while intra-cycle (within menstrual cycle) reliability was sufficient, we found insufficient inner-cycle (between menstrual cycles) reliability. Concerning inner-cycle reliability, it might be argued that the 60 days between baseline and treatment cycle 2 may have been too long, and that the results for intra-cycle reliability can be considered more indicative for the true reliability. For this reason, we decided not to consider the insufficient reliability between menstrual cycles when deriving a recommendation for use, but stress that sufficient reliability of the DysDD is only given when administered within the menstrual cycle. Regarding aspects of feasibility, the DysDD was administered as eDiary in the validation study, suggesting that the tool has the potential to be used by physicians in daily practice and by researchers in studies involving women with PDys. Underlining the usefulness of the DysDD, data on MID indicate that a change of three points in the pelvic pain score can be considered clinically meaningful.

Taken together, our evaluation revealed that the ADSCS can be recommended as PROM for the assessment of self-care behavior of adolescent girls with PDys, but requires further content validity assessments. Regarding measures of impact, the on-menses version of the DSI can be recommended for use, while the DysDD does currently not fulfill the COSMIN criteria for a recommendation. However, given the intensive work on scale development and testing during the PROM design phase and the broad spectrum of outcomes covered, the DysDD appears promising for use in medical care and research, encouraging further investigations. Overall, the insufficient construct validity of the DSI off-menses version and the insufficient intra-cycle reliability of the DysDD indicate that recalling PDys symptoms and associated impairment referring to the last menstrual period may result in invalid data. Along with the finding that construct validity of the DSI on-menses version and intra-cycle reliability of the DysDD were sufficient, the present data strongly suggest that measures of PDys should refer to the current menstrual cycle with daily monitoring of symptoms and impact.

The results of the present systematic review provide important implications for use of the identified instruments in patient care and research. For the measurement of self-care behavior, the ADSCS is a suitable instrument. Helping to identify counseling needs and to offer appropriate support, this instrument can be recommended for use in care for adolescent girls with PDys. The on-menses of the DSI can be recommended for disease monitoring and for the evaluation of the effectiveness of treatments from the patients’ perspective, which is relevant for both patient care and research. For this purpose, also the DysDD including daily assessments might be a suitable tool, but requires further validation.

### Strengths and limitations

Strengths of the present work encompass the application of an established comprehensive and sensitive search filter, which was not restricted to publication year and language. Allowing to capture all potentially relevant outcomes, our search strategy included any PROMs for women with PDys. Our literature search was carried out in the two major databases PubMed and Web of Science, and we additionally searched the reference lists of the included studies for relevant articles. Moreover, we contacted the authors of the included studies to obtain further information on research activities regarding PROMs for PDys. Notably, due to the methodology applied in the present systematic review, only PROMs for which validation studies were available could be considered. A limitation may arise from the fact that we did not search all reference lists of relevant full texts for further eligible studies, and that further databases such as Scopus, Embase, or PsycINFO were not considered. However, in the biomedical field, PubMed is considered the leading database [26].

## Conclusions

We identified four PROMs for use in women with PDys focusing on various outcomes. According to COSMIN criteria, the ADSCS can be recommended for the assessment of self-care behavior of adolescent girls with PDys. To measure the impact of PDys symptoms on the women's daily activities, the on-menses version of the DSI can be recommended. Although both instruments showed sufficient content validity, major shortcomings concern the deficient patient involvement in the content validity study of the ADSCS, and the lack of patient engagement in the design of the DSI, indicating the need for further content validity studies. Applying the criteria of the FDA for the evaluation of PROMs, which require patient involvement in the item generation phase [25], the DSI would not be accepted as measure for endpoints in clinical trials. The DysDD has the potential to be recommended for use, but further validation studies assessing content validity and structural validity are required.

### Supplementary Information

Below is the link to the electronic supplementary material.

Supplementary file1 (DOCX 68 KB)

Supplementary file2 (DOCX 48 KB)

Supplementary file3 (DOCX 22 KB)

Supplementary file4 (DOCX 19 KB)

Supplementary file5 (DOCX 47 KB)

Supplementary file6 (DOCX 48 KB)

Supplementary file7 (DOCX 48 KB)

## Data Availability

The articles used and analyzed during the current study are available from the corresponding author on reasonable request.

## Code availability

All data analyses were conducted using the COSMIN manuals (available at https://www.cosmin.nl/tools/guideline-conducting-systematic-review-outcome-measures/).

## References

[1] Burnett M, Lemyre M (2017). No 345-primary dysmenorrhea consensus guideline. Journal of Obstetrics and Gynaecology Canada: JOGC = Journal d'obstetrique et gynecologie du Canada: JOGC.

[2] Iacovides S, Avidon I, Baker FC (2015). What we know about primary dysmenorrhea today: A critical review. Human reproduction update.

[3] Ju H, Jones M, Mishra G (2014). The prevalence and risk factors of dysmenorrhea. Epidemiologic Reviews.

[4] Itani R, Soubra L, Karout S, Rahme D, Karout L, Khojah HMJ (2022). Primary dysmenorrhea: Pathophysiology, diagnosis, and treatment updates. Korean Journal of Family Medicine.

[5] Sharghi M, Mansurkhani SM, Larky DA, Kooti W, Niksefat M, Firoozbakht M (2019). An update and systematic review on the treatment of primary dysmenorrhea. JBRA Assisted Reproduction.

[6] Churruca K, Pomare C, Ellis LA, Long JC, Henderson SB, Murphy LED (2021). Patient-reported outcome measures (PROMs): A review of generic and condition-specific measures and a discussion of trends and issues. Health Expectations: An International Journal of Public Participation in Health Care and Health Policy.

[7] Prinsen CAC, Mokkink LB, Bouter LM, Alonso J, Patrick DL, de Vet HCW (2018). COSMIN guideline for systematic reviews of patient-reported outcome measures. Quality of Life Research: An International Journal of Quality of Life Aspects of Treatment, Care and Rehabilitation.

[8] Mokkink LB, Terwee CB, Patrick DL, Alonso J, Stratford PW, Knol DL (2010). The COSMIN study reached international consensus on taxonomy, terminology, and definitions of measurement properties for health-related patient-reported outcomes. Journal of Clinical Epidemiology.

[9] Mokkink LB, Terwee CB, Knol DL, Stratford PW, Alonso J, Patrick DL (2010). The COSMIN checklist for evaluating the methodological quality of studies on measurement properties: A clarification of its content. BMC Medical Research Methodology.

[10] Page MJ, McKenzie JE, Bossuyt PM, Boutron I, Hoffmann TC, Mulrow CD (2021). The PRISMA 2020 statement: An updated guideline for reporting systematic reviews. Systematic Reviews.

[11] Mokkink LB, de Vet HCW, Prinsen CAC, Patrick DL, Alonso J, Bouter LM (2018). COSMIN Risk of Bias checklist for systematic reviews of Patient-Reported Outcome Measures. Quality of Life Research: An International Journal of Quality of Life Aspects of Treatment, Care and Rehabilitation.

[12] Ouzzani M, Hammady H, Fedorowicz Z, Elmagarmid A (2016). Rayyan-a web and mobile app for systematic reviews. Systematic Reviews.

[13] Terwee CB, Prinsen CAC, Chiarotto A, Westerman MJ, Patrick DL, Alonso J, Bouter LM, de Vet HCW, Mokkink LB (2018). COSMIN methodology for evaluating the content validity of patient-reported outcome measures: a Delphi study. Quality of Life Research: An International Journal of Quality of Life Aspects of Treatment, Care and Rehabilitation.

[14] Mokkink LB, Prinsen CA, Patrick DL, Alonso J, Bouter LM, De Vet HC, et al. COSMIN methodology for systematic reviews of Patient-Reported Outcome Measures (PROMs)—user manual. https://cosmin.nl/wp-content/uploads/COSMIN-syst-review-for-PROMs-manual_version-1_feb-2018.pdf 10.1007/s11136-018-1798-3 PMC5891568 29435801

[15] Nguyen AM, Humphrey L, Kitchen H, Rehman T, Norquist JM (2015). A qualitative study to develop a patient-reported outcome for dysmenorrhea. Quality of Life Research: An International Journal of Quality of Life Aspects of Treatment, Care and Rehabilitation.

[16] Nguyen AM, Arbuckle R, Korver T, Chen F, Taylor B, Turnbull A (2017). Psychometric validation of the dysmenorrhea daily diary (DysDD): A patient-reported outcome for dysmenorrhea. Quality of Life Research: An International Journal of Quality of Life Aspects of Treatment, Care and Rehabilitation.

[17] Wong CL, Ip WY, Shiu TY (2012). Translation and validation of the Chinese-Cantonese version of the exercise of self-care agency scale. International Journal of Nursing Studies.

[18] Wong CL, Ip WY, Choi KC, Shiu TY (2013). Translation and validation of the Chinese-Cantonese version of the adolescent dysmenorrhic self-care scale in Hong Kong adolescent girls. Journal of Clinical Nursing.

[19] Chen CX, Murphy T, Ofner S, Yahng L, Krombach P, LaPradd M (2021). Development and testing of the dysmenorrhea symptom interference (DSI) scale. Western Journal of Nursing Research.

[20] Kearney BY, Fleischer BJ (1979). Development of an instrument to measure exercise of self-care agency. Research in Nursing & Health.

[21] Ching-Hsing H, Meei-Ling G, Hsin-Chun M, Chung-Yi L (2004). The development and psychometric testing of a self-care scale for dysmenorrhic adolescents. The Journal of Nursing Research: JNR.

[22] Chen CX, Kwekkeboom KL, Ward SE (2015). Self-report pain and symptom measures for primary dysmenorrhoea: A critical review. European Journal of Pain (London, England).

[23] Hartweg DL (1995). Dorothea orem: Self-care deficit theory (notes on nursing theories, Vol. 4).

[24] Wiering B, de Boer D, Delnoij D (2017). Patient involvement in the development of patient-reported outcome measures: The developers' perspective. BMC Health Services Research.

[25] U.S. Department of Health and Human Services FDA Center for Drug Evaluation and Research; U.S. Department of Health and Human Services FDA Center for Biologics Evaluation and Research; U.S. Department of Health and Human Services FDA Center for Devices and Radiological Health. (2006). Guidance for industry: Patient-reported outcome measures: Use in medical product development to support labeling claims: Draft guidance. *Health and Quality of Life Outcomes,**4*, 79. 10.1186/1477-7525-4-79. 10.1186/1477-7525-4-79 PMC1629006 17034633

[26] Falagas ME, Pitsouni EI, Malietzis GA, Pappas G (2008). Comparison of PubMed, scopus, web of science, and google scholar: Strengths and weaknesses. FASEB Journal: Official Publication of the Federation of American Societies for Experimental Biology.

