# Supplementary material for: Quality of patient-reported outcome measures for primary dysmenorrhea: a systematic review
Source: Qual Life Res. 2023 Oct 30;33(1):31–43. doi: 10.1007/s11136-023-03517-8 (PMC10784326; doi:10.1007/s11136-023-03517-8)
Supplement: Supplementary file 1 — Supplementary file1 (DOCX 68 KB) [file 11136_2023_3517_MOESM1_ESM.docx]

**Appendix 1** Literature search

The search terms to define the target population were developed by the review team in collaboration with a physician and a biologist with expertise regarding womens’ gynecological conditions. To assess construct, measurement properties, feasibility as well as to create an exclusion filter, we applied filters recommended by the COSMIN group. We further adapted established and previously published filters. For search in Web of Science, we adapted the search strategy for PubMed using appropriate syntax and index terms. Our search was not restricted to publication date and language.

The search strategy comprised the following elements:

*A. Target population:* Women with PDys. We used a comprehensive compilation of controlled vocabulary and free text terms based on the literature to allow for a broad sensitivity. In order not to miss any potentially relevant studies, we included all possible spellings for the terms related to PDys.

*B. Construct of interest:* All PROMs related to PDys. The search for this element is based on a combination of the PubMed filter "Quality of life (QoL)" of Vissers and de Vries [1], the PubMed filter "Patient reported outcome measures (PROMs)" of Jansma and de Vries [2], and on additional search terms from the filter "PROM group construct & instrument type filter" of Mackintosh et al. [3].

*C. Measurement properties:* We used the validated and sensitive search filter for PubMed by Terwee et al. [4].

*D. Feasibility of PROMs:* This element was based on the search terms for the concept ‘feasibility’ of Heinl et al. (included in their search statement #1, Additional file 2) [5].

*E. Individual PROMs:* A list of PROMs in the context of PDys already known was included.

*F. Exclusion filter:* We applied the filter by Terwee et al. [4] to exclude irrelevant publication types and animal studies. We further excluded disease conditions related to secondary dysmenorrhea (chronic pelvic pain, chronic pelvic pain syndrome, endometriosis, fibroids, uterine adenomyosis).

The single search elements were combined as follows: (((A AND B AND (C OR D)) OR (C AND E)) NOT F); in words: (((population AND construct AND (measurement properties OR feasibility)) OR (individual PROMs AND measurement properties)) NOT (exclusion filter)).

Search strategy for PUBMED

**### A: Target population – Primary dysmenorrhea**

(“dysmenorrhea“ [Title/Abstract] OR “dysmenorrhoea“ [Title/Abstract] OR “primary dysmenorrhea“ [Title/Abstract] OR “primary dysmenorrhoea“ [Title/Abstract] OR “menstrual cramps“ [Title/Abstract] OR “menstrual pain“ [Title/Abstract] OR “period pain“ [Title/Abstract] OR “painful periods“ [Title/Abstract] OR “painful menstrual periods“ [Title/Abstract] OR “painful menstruation“ [Title/Abstract] OR “menorrhalgia“ [Title/Abstract] OR “cramping pain“ [Title/Abstract])

**### B: Construct – PROMs including Quality of Life**

(“Patient Reported Outcome Measures”[Mesh] OR “Quality of Life”[Mesh] OR prom[tiab] OR proms[tiab] OR pro[tiab] OR pros[tiab] OR HRQL[tiab] OR HRQoL[tiab] OR QL[tiab] OR QoL[tiab] OR quality of life[tiab] OR life quality[tiab] OR health index*[tiab] OR health indices[tiab] OR health profile*[tiab] OR health status[tw] OR ((patient[tiab] OR self[tiab] OR carer[tiab] OR proxy[tiab]) AND ((report[tiab] OR reported[tiab] OR reporting[tiab]) OR (rated[tiab] OR rating[tiab] OR ratings[tiab]) OR based[tiab] OR (assessed[tiab] OR assessment[tiab] OR assessments[tiab]))) OR ((disability[tiab] OR function[tiab] OR functional[tiab] OR functions[tiab] OR subjective[tiab] OR utility[tiab] OR utilities[tiab] OR wellbeing[tiab] OR well being[tiab]) AND (outcome[tiab] OR outcomes[tiab] OR index[tiab] OR indices[tiab] OR instrument[tiab] OR instruments[tiab] OR measure[tiab] OR measures[tiab] OR questionnaire[tiab] OR questionnaires[tiab] OR profile[tiab] OR profiles[tiab] OR scale[tiab] OR scales[tiab] OR score[tiab] OR scores[tiab] OR status[tiab] OR survey[tiab] OR surveys[tiab])))

**### C: Measurement properties**

(instrumentation[sh] OR methods[sh] OR “Comparative Study”[pt] OR “psychometrics”[MeSH] OR psychometr*[tiab] OR clinimetr*[tw] OR clinometr*[tw] OR “outcome assessment”[tiab] OR “outcome measure*”[tw] OR “observer variation”[MeSH] OR “observer variation”[tiab] OR “Health Status Indicators”[Mesh] OR “reproducibility of results”[MeSH] OR reproducib*[tiab] OR “discriminant analysis”[MeSH] OR reliab*[tiab] OR unreliab*[tiab] OR valid*[tiab] OR “coefficient of variation”[tiab] OR coefficient[tiab] OR homogeneity[tiab] OR homogeneous[tiab] OR “internal consistency”[tiab] OR (cronbach*[tiab] AND (alpha[tiab] OR alphas[tiab])) OR (item[tiab] AND (correlation*[tiab] OR selection*[tiab] OR reduction*[tiab])) OR agreement[tw] OR precision[tw] OR imprecision[tw] OR “precise values”[tw] OR test-retest[tiab] OR (test[tiab] AND retest[tiab]) OR (reliab*[tiab] AND (test[tiab] OR retest[tiab])) OR stability[tiab] OR interrater[tiab] OR inter-rater[tiab] OR intrarater[tiab] OR intra-rater[tiab] OR intertester[tiab] OR inter-tester[tiab] OR intratester[tiab] OR intra-tester[tiab] OR interobserver[tiab] OR inter-observer[tiab] OR intraobserver[tiab] OR intra-observer[tiab] OR intertechnician[tiab] OR inter-technician[tiab] OR intratechnician[tiab] OR intra-technician[tiab] OR interexaminer[tiab] OR inter-examiner[tiab] OR intraexaminer[tiab] OR intra-examiner[tiab] OR interassay[tiab] OR inter-assay[tiab] OR intraassay[tiab] OR intra-assay[tiab] OR interindividual[tiab] OR inter-individual[tiab] OR intraindividual[tiab] OR intra-individual[tiab] OR interparticipant[tiab] OR inter-participant[tiab] OR intraparticipant[tiab] OR intra-participant[tiab] OR kappa[tiab] OR kappa’s[tiab] OR kappas[tiab] OR repeatab*[tw] OR ((replicab*[tw] OR repeated[tw]) AND (measure[tw] OR measures[tw] OR findings[tw] OR result[tw] OR results[tw] OR test[tw] OR tests[tw])) OR generaliza*[tiab] OR generalisa*[tiab] OR concordance[tiab] OR (intraclass[tiab] AND correlation*[tiab]) OR discriminative[tiab] OR “known group”[tiab] OR “factor analysis”[tiab] OR “factor analyses”[tiab] OR “factor structure”[tiab] OR “factor structures”[tiab] OR dimension*[tiab] OR subscale*[tiab] OR (multitrait[tiab] AND scaling[tiab] AND (analysis[tiab] OR analyses[tiab])) OR “item discriminant”[tiab] OR “interscale correlation*”[tiab] OR error[tiab] OR errors[tiab] OR “individual variability”[tiab] OR “interval variability”[tiab] OR “rate variability”[tiab] OR (variability[tiab] AND (analysis[tiab] OR values[tiab])) OR (uncertainty[tiab] AND (measurement[tiab] OR measuring[tiab])) OR “standard error of measurement”[tiab] OR sensitiv*[tiab] OR responsive*[tiab] OR (limit[tiab] AND detection[tiab]) OR “minimal detectable concentration”[tiab] OR interpretab*[tiab] OR ((minimal[tiab] OR minimally[tiab] OR clinical[tiab] OR clinically[tiab]) AND (important[tiab] OR significant[tiab] OR detectable[tiab]) AND (change[tiab] OR difference[tiab])) OR (small*[tiab] AND (real[tiab] OR detectable[tiab]) AND (change[tiab] OR difference[tiab])) OR “meaningful change”[tiab] OR “ceiling effect”[tiab] OR “floor effect”[tiab] OR “Item response model”[tiab] OR IRT[tiab] OR Rasch[tiab] OR “Differential item functioning”[tiab] OR DIF[tiab] OR “computer adaptive testing”[tiab] OR “item bank”[tiab] OR “cross-cultural equivalence”[tiab])

**### D: Feasibility of PROMs**

(((accepta*[Title/Abstract]) OR (“ease of use”[Title/Abstract])) OR (practica*[Title/Abstract])) OR (feasib*[Title/Abstract])

**### E: Known PROMs**

(“DysDD”[Title/Abstract] OR (“dysmenorrhea “[Title/Abstract] AND “diary”[Title/Abstract]))

**### F: Exclusion filter**

(“addresses“ OR “biography“ OR “case reports“ OR “comment“ OR “directory“ OR “editorial“ OR “festschrift“ OR “interview“ OR “lectures“ OR “legal cases“ OR “legislation“ OR “letter“ OR “news“ OR “newspaper article“ OR “patient education handout“ OR “popular works“ OR “congresses“ OR “consensus development conference“ OR “consensus development conference, nih“ OR “practice guideline“) OR (exp “animals“/ NOT “humans”) OR “secondary dysmenorrhea” OR (“secondary dysmenorrhoea” OR “chronic pelvic pain” OR “chronic pelvic pain syndrome” OR “endometriosis” OR “fibroids” OR “uterine adenomyosis“)

References

[1] Vissers T, Vries R. D. Quality of life (QoL) search block. [June 30, 2023]; Available from: https://blocks.bmi-online.nl/catalog/294.

[2] Jansma EP, Vries R. D. Patient reported outcome measures (PROMs) search block. [June 30, 2023].

[3] Mackintosh A, Comabella CCI, Hadi M, et al. PROM group construct & instrument type filters. [June 30, 2023].

[4] Terwee CB, Jansma EP, Riphagen II, Vet HCW de. Development of a methodological PubMed search filter for finding studies on measurement properties of measurement instruments. Qual Life Res 2009;18(8):1115–23. https://doi.org/10.1007/s11136-009-9528-5.

[5] Heinl D, Prinsen CAC, Drucker AM, Ofenloch R, Humphreys R, Sach T et al. Measurement properties of quality of life measurement instruments for infants, children and adolescents with eczema: protocol for a systematic review. Syst Rev 2016;5:25. https://doi.org/10.1186/s13643-016-0202-z.
