# Supplementary material for: Quality of patient-reported outcome measures for primary dysmenorrhea: a systematic review
Source: Qual Life Res. 2023 Oct 30;33(1):31–43. doi: 10.1007/s11136-023-03517-8 (PMC10784326; doi:10.1007/s11136-023-03517-8)
Supplement: Supplementary file 2 — Supplementary file2 (DOCX 48 KB) [file 11136_2023_3517_MOESM2_ESM.docx]

**Appendix 2** Boxes of the COSMIN Risk of Bias checklist

| **Content validity** | |
| --- | --- |
| Box 1 | PROM development |
| Box 2 | Content validity |
| **Internal structure** | |
| Box 3 | Structural validity |
| Box 4 | Internal consistency |
| **Remaining measurement properties** | |
| Box 5 | Cross-cultural validity/measurement invariance |
| Box 6 | Reliability |
| Box 7 | Measurement error |
| Box 8 | Criterion validity |
| Box 9 | Hypotheses testing for construct validity |
| Box 10 | Responsiveness |

*COSMIN* COnsensus‐based Standards for the selection of health Measurement INstruments,
*PROM* patient-reported outcome measure
