# Supplementary material for: Quality of patient-reported outcome measures for primary dysmenorrhea: a systematic review
Source: Qual Life Res. 2023 Oct 30;33(1):31–43. doi: 10.1007/s11136-023-03517-8 (PMC10784326; doi:10.1007/s11136-023-03517-8)
Supplement: Supplementary file 3 — Supplementary file3 (DOCX 22 KB) [file 11136_2023_3517_MOESM3_ESM.docx]

**Appendix 3** Criteria for good measurement properties

| **Measurement property** | **Rating** | **Criteria** |
| --- | --- | --- |
| **Structural validity** | + | **CTT**  CFA: CFI or comparable measure > 0.95 OR RMSEA < 0.06 OR  SRMR < 0.08^a^  **IRT/Rasch**  No violation of unidimensionality^b^: CFI or TLI or comparable measure > 0.95 OR RMSEA < 0.06 OR SRMR < 0.08  *AND*  no violation of local independence: residual correlations among the items after controlling for the dominant factor < 0.20 OR Q3’s < 0.37  *AND*  no violation of monotonicity: adequate looking graphs OR item scalability > 0.30  *AND*  adequate model fit  IRT: χ^2^ > 0.001  Rasch: infit and outfit mean squares ≥ 0.5 and ≤ 1.5 OR Z-standardized values > -2 and < 2 |
|  | ? | CTT: not all information for ‘+’ reported  IRT/Rasch: model fit not reported |
|  | - | Criteria for ‘+’ not met |
| **Internal consistency** | + | At least low evidence^c^ for sufficient structural validity^d^” AND Cronbach’s alpha(s) ≥ 0.70 for each unidimensional scale or subscale^e^ |
|  | ? | Criteria for “At least low evidence^c^ for sufficient structural validity^d^” not met |
|  | - | Cronbach’s alpha(s) < 0.70 for each unidimensional scale or subscale^e^ |
| **Cross-cultural validity/ measurement invariance** | + | No important differences found between group factors (such as age, gender, language) in multiple group factor analysis OR no important DIF for group factors (McFadden’s R^2^ < 0.02) |
|  | ? | No multiple group factor analysis OR DIF analysis performed |
|  | - | Important differences between group factors OR DIF was found |
| **Reliability** | + | ICC or weighted Kappa ≥ 0.70 |
|  | ? | ICC or weighted Kappa not reported |
|  | - | ICC or weighted Kappa < 0.70 |
| **Measurement error** | + | SDC or LoA < MIC^d^ |
|  | ? | MIC not defined |
|  | - | SDC or LoA > MIC^d^ |
| **Criterion validity** | + | Correlation with gold standard ≥ 0.70 OR AUC ≥ 0.70 |
|  | ? | Not all information for ‘+’ reported |
|  | - | Correlation with gold standard < 0.70 OR AUC < 0.70 |

| **Hypotheses testing for construct validity** | + | The result is in accordance with the hypothesis^f^ |
| --- | --- | --- |
|  | ? | No hypothesis defined (by the review team) |
|  | - | The result is not in accordance with the hypothesis^f^ |
| **Responsiveness** | + | The result is in accordance with the hypothesis^f^ OR AUC ≥ 0.70 |
|  | ? | No hypothesis defined (by the review team) |
|  | - | The result is not in accordance with the hypothesis^f^ OR AUC < 0.70 |

*AUC* area under the curve, *CFA* confirmatory factor analysis, *CFI* comparative fit index, *CTT* classical test theory,
*DIF* differential item functioning, *ICC* intraclass correlation coefficient, *IRT* Item response theory, *LoA* limits of agreement, *MIC* minimal important change, *RMSEA* root mean square error of approximation, *SEM* standard error of measurement, *SDC* smallest detectable change, *SRMR* standardized root mean residuals, *TLI* Tucker-Lewis index

“+” = sufficient, “-“ = insufficient, “?” = indeterminate

^a^To rate the quality of the summary score, the factor structure should be equal across studies.

^b^Unidimensionality refers to a factor analysis per subscale, while structural validity refers to a factor analysis of a (multidimensional) patient-reported outcome measure.

^c^As defined by grading the evidence according to the GRADE approach.

^d^This evidence may come from different studies.

^e^The criteria ‘Cronbach’s alpha < 0.95’ was deleted, as this is relevant in the development phase of a PROM and not when evaluating an existing PROM.

^f^The results of all studies should be taken together and it should then be decided if 75% of the results are in accordance with the hypotheses.
