# Supplementary material for: Quality of patient-reported outcome measures for primary dysmenorrhea: a systematic review
Source: Qual Life Res. 2023 Oct 30;33(1):31–43. doi: 10.1007/s11136-023-03517-8 (PMC10784326; doi:10.1007/s11136-023-03517-8)
Supplement: Supplementary file 4 — Supplementary file4 (DOCX 19 KB) [file 11136_2023_3517_MOESM4_ESM.docx]

**Appendix 4** Excluded instruments

| **Instrument** | **Reference** | **Purpose** | **Reason for exclusion** |
| --- | --- | --- | --- |
| **Menstrual Symptom Questionnaire  (MSQ)** | Chestney and Tasto 1975^a^ | To differentiate between spasmodic and congestive dysmenorrhea. | Since the development of the MSQ,  the understanding and definition of menstrual symptoms has evolved, and evidence suggests that this tool can no longer be assumed to measure dysmenorrhea.^e^ |
| **Verbal Multidimensional Scoring Assessment for Dysmenorrhea  (VMS)** | Andersch and Milsom 1982^b^ | To measure the severity of primary dysmenorrhea using four categories considering working ability, systemic symptoms and the use of analgesics. | No validation studies are available. |
| **Retrospective Symptom Scale  (RSS)** | Cox and Meyer 1978^c^ | To measure frequency and severity of commonly reported physical and emotional dysmenorrhea symptoms, invalid hours, and units of medication used referring to the last menstrual period. | The RSS and DSS were developed and administered in a study investigating behavioral treatment parameters in women with primary dysmenorrhea.  No validation studies are available. |
| **Daily Symptom Scale  (DSS)** | Cox and Meyer 1978^c^ | To measure frequency and severity of commonly reported physical and emotional dysmenorrhea symptoms, invalid hours, and units of medication as daily diary. |  |
| **Symptom Severity Scale  (SSS)** | Chestney and Tasto 1975^d^ | To measure the degree to which women experience discomfort during their last menstrual period. | The SSS was developed and administered in a study testing the effectiveness of behavior modification in women with spasmodic and congestive dysmenorrhea. No validation studies are available. |

^a^Chesney, M. A., & Tasto, D. L. (1975). The development of the menstrual symptom questionnaire. Behaviour research and therapy, 13(4), 237–244.

^b^Andersch, B., & Milsom, I. (1982). An epidemiologic study of young women with dysmenorrhea. *American journal of obstetrics and gynecology*, 144(6), 655–660.

^c^Cox, D. J., & Meyer, R. G. (1978). Behavioral treatment parameters with primary dysmenorrhea. *Journal of behavioral medicine*, 1(3), 297–310.

^d^Chesney, M. A., & Tasto, D. L. (1975). The effectiveness of behavior modification with spasmodic and congestive dysmenorrhea. *Behaviour research and therapy*, 13(4), 245–253.

^e^Negriff, S., Dorn, L. D., Hillman, J. B., & Huang, B. (2009). The measurement of menstrual symptoms: factor structure of the menstrual symptom questionnaire in adolescent girls. *Journal of health psychology*, 14(7), 899–908.
