# Supplementary material for: Quality of patient-reported outcome measures for primary dysmenorrhea: a systematic review
Source: Qual Life Res. 2023 Oct 30;33(1):31–43. doi: 10.1007/s11136-023-03517-8 (PMC10784326; doi:10.1007/s11136-023-03517-8)
Supplement: Supplementary file 5 — Supplementary file5 (DOCX 47 KB) [file 11136_2023_3517_MOESM5_ESM.docx]

**Appendix 5** Availability of the identified instruments

**Exercise of Self-Care Agency Scale (ESCAS)**

The English and the Chinese-Cantonese version of the ESCAS is presented in the research paper: https://pubmed.ncbi.nlm.nih.gov/22572019/

**Adolescent Dysmenorrhic Self-Care Scale (ADSCS)**

The English version of the ADSCS is presented in the research paper: https://pubmed.ncbi.nlm.nih.gov/23228020/

**Dysmenorrhea Symptom Interference Scale (DSI)**

The English version of the DSI can be accessed online via the [Mapi Research Trust](https://mapi-trust.org/) online platform ePROVIDE: https://eprovide.mapi-trust.org/instruments/dysmenorrhea-symptom-interference-scale

**Dysmenorrhea Daily Diary (DysDD)**

The English version of the DysDD can be accessed online via the [Mapi Research Trust](https://mapi-trust.org/) online platform ePROVIDE: https://eprovide.mapi-trust.org/instruments/dysmenorrhea-daily-diary
