# Supplementary material for: Quality of patient-reported outcome measures for primary dysmenorrhea: a systematic review
Source: Qual Life Res. 2023 Oct 30;33(1):31–43. doi: 10.1007/s11136-023-03517-8 (PMC10784326; doi:10.1007/s11136-023-03517-8)
Supplement: Supplementary file 6 — Supplementary file6 (DOCX 48 KB) [file 11136_2023_3517_MOESM6_ESM.docx]

**Appendix 6** COSMIN Risk of Bias overall ratings of PROM development and content validity studies

|  | **ESCAS** | **ADSCS** | **DSI** | **DysDD** |
| --- | --- | --- | --- | --- |
| **Box 1. PROM development** | Inadequate^a^ | Doubtful^c^ | Inadequate^e^ | Doubtful^f^ |
| **Box 2. Content validity** | Doubtful^b^ | Doubtful^d^ | Doubtful^e^ | **-** |

*COSMIN* COnsensus‐based Standards for the selection of health Measurement INstruments, *PROM* patient-reported outcome measure

*ADSCS* Adolescent Dysmenorrhic Self-Care Scale, *ESCAS* Exercise of Self-Care Agency Scale, *DSI* Dysmenorrhea Symptom Interference Scale, *DysDD* Dysmenorrhea Daily Diary

^a^Kearney and Fleischer 1979

^b^Wong et al. 2012a

^c^Hsieh et al. 2004

^d^Wong et al. 2012b

^e^Chen et al. 2021

^f^Nguyen et al. 2015
