# Supplementary material for: Quality of patient-reported outcome measures for primary dysmenorrhea: a systematic review
Source: Qual Life Res. 2023 Oct 30;33(1):31–43. doi: 10.1007/s11136-023-03517-8 (PMC10784326; doi:10.1007/s11136-023-03517-8)
Supplement: Supplementary file 7 — Supplementary file7 (DOCX 48 KB) [file 11136_2023_3517_MOESM7_ESM.docx]

**Appendix 7** Content validity rating of the included instruments

|  |  | **Relevance** | **Comprehensiveness** | **Comprehensibility** | **Content validity rating** |
| --- | --- | --- | --- | --- | --- |
| **ESCAS** | Overall rating | ? | ? | ? | Indeterminate (?) |
|  | Quality of evidence | No grading if overall rating is indeterminate | | |  |
| **ADSCS** | Overall rating | + | + | + | Sufficient (+) |
|  | Quality of evidence | Moderate (due to risk of bias) | | | |
| **DSI** | Overall rating | + | + | + | Sufficient (+) |
|  | Quality of evidence | Moderate (due to risk of bias) | | | |
| **DysDD** | Overall rating | + | + | + | Sufficient (+) |
|  | Quality of evidence | Low (due to risk of bias) | | | |

*ADSCS* Adolescent Dysmenorrhic Self-Care Scale, *ESCAS* Exercise of Self-Care Agency Scale, *DSI* Dysmenorrhea Symptom Interference Scale,
*DysDD* Dysmenorrhea Daily Diary
